# Time to revisit the passive overconsumption hypothesis? Humans show sensitivity to calories in energy-rich meals

**DOI:** 10.1093/ajcn/nqac112

**Published:** 2022-04-30

**Authors:** Annika N Flynn, Kevin D Hall, Amber B Courville, Peter J Rogers, Jeffrey M Brunstrom

**Affiliations:** Nutrition and Behaviour Unit, School of Psychological Science, University of Bristol, Bristol, United Kingdom; National Institute of Diabetes and Digestive and Kidney Diseases, Bethesda, MD, USA; National Institute of Diabetes and Digestive and Kidney Diseases, Bethesda, MD, USA; Nutrition and Behaviour Unit, School of Psychological Science, University of Bristol, Bristol, United Kingdom; National Institute for Health and Care Research (NIHR) Bristol Biomedical Research Centre: Nutrition Theme, University of Bristol, University Hospitals Bristol Education & Research Centre, Bristol, United Kingdom; Nutrition and Behaviour Unit, School of Psychological Science, University of Bristol, Bristol, United Kingdom; National Institute for Health and Care Research (NIHR) Bristol Biomedical Research Centre: Nutrition Theme, University of Bristol, University Hospitals Bristol Education & Research Centre, Bristol, United Kingdom

**Keywords:** energy density, energy intake, passive overconsumption, meal size, volume, calorie content, energy balance, satiation, satiety, ultra-processing

## Abstract

**Background:**

A possible driver of obesity is insensitivity (passive overconsumption) to food energy density (ED, kcal/g); however, it is unclear whether this insensitivity applies to all meals.

**Objectives:**

We assessed the influence of ED on energy intake (kcal) across a broad and continuous range of EDs comprised of noncovertly manipulated, real-world meals. We also allowed for the possibility that the association between energy intake and ED is nonlinear.

**Methods:**

We completed a secondary analysis of 1519 meals which occurred in a controlled environment as part of a study conducted by Hall and colleagues to assess the effects of food ultra-processing on energy intake. To establish the generalizability of the findings, the analyses were repeated in 32,162 meals collected from free-living humans using data from the UK National Diet and Nutrition Survey (NDNS). Segmented regressions were performed to establish ED “breakpoints” at which the association between consumed meal ED and mean centered meal caloric intake (kcal) changed.

**Results:**

Significant breakpoints were found in both the Hall et al. data set (1.41 kcal/g) and the NDNS data set (1.75 and 2.94 kcal/g). Centered meal caloric intake did not increase linearly with consumed meal ED, and this pattern was captured by a 2-component (“volume” and “calorie content” [biologically derived from the sensing of fat, carbohydrate, and protein]) model of physical meal size (g), in which volume is the dominant signal with lower energy-dense foods and calorie content is the dominant signal with higher energy-dense foods.

**Conclusions:**

These analyses reveal that, on some level, humans are sensitive to the energy content of meals and adjust meal size to minimize the acute aversive effects of overconsumption. Future research should consider the relative importance of volume and calorie-content signals, and how individual differences impact everyday dietary behavior and energy balance.

## Introduction

Food energy density (ED, kcal/g) refers to the energy content (kcal) of a specified weight of food (g) and can differ considerably between foods. For example, cucumber and pecan nuts have an ED of 0.15 kcal/g and 7.26 kcal/g, respectively. From the early 1980s, the literature has linked excess energy intake (kcal) to an inability to “compensate” for differences in meal ED by selecting smaller meals with increasing ED (1–3). Two main methodologies, *1*) energy intake during ad libitum meals (4) and *2*) test meals following a food or beverage preload (preload test-meal paradigm) (4), assess the effects of ED on energy intake via compensatory changes in meal size. However, these 2 methods produce different findings regarding sensitivity to food energy content.

With respect to satiation, many well-designed ad libitum meal studies find little or no sensitivity to ED within a meal (i.e. the same weight of food is consumed irrespective of ED). In most of these studies, ED was covertly manipulated over a short period of time (e.g. <10 total exposure days) (1–3, 5, 6). In other words, differences in meal caloric content have little impact on the amount of food ingested, and this insensitivity can persist over several days (2, 3). This effect of ED on energy intake, particularly in the case of high-fat food consumption, is sometimes referred to as “passive overconsumption” (7).

By contrast, studies of satiety (preload test-meal paradigm) provide strong evidence that calories in a preload can influence subsequent energy intake, highlighting a variable degree of short-term compensation in response to covertly manipulating the preload ED, even including sugar in a beverage (8–14). These studies demonstrate that calories can influence behavior and subsequent food intake after a meal has ended. Furthermore, partial compensation to the manipulation of ED is found in ad libitum studies that expose participants to covertly manipulated diets over long periods of time (e.g. >10 total exposure days) (15–17). Compensation in these various studies represents an “unlearned” response to the satiating effect of calories (single or first exposure) (9, 18) as well as a potentially learned response (repeated exposure) (9, 18–21).

In relation to these apparently contradictory findings and a separate finding of a nonlinear relation between absolute ED and its effect on behavior, specifically portion size selection (22), we set out to re-evaluate the association between meal ED and meal energy intake. Importantly, we did this using a broad and continuous range of EDs and “real-world” foods, rather than covertly manipulated test meals.

We analyzed data from a recent study investigating the effects of ultra-processing on energy intake over time and under controlled conditions (23), and assessed whether the association between energy intake and ED is nonlinear. To investigate the generalizability of these findings, we then performed the same analysis on data collected from free-living humans in the UK (24). Based on our results, we developed a unifying “2-component model” which explains the previous findings from ad libitum and preload test-meal studies and the relation we observe between ED and energy intake.

## Methods

### Overview of the Hall et al. study on food ultra-processing and energy intake

Hall et al. (23) assessed a potential causal association between the consumption of ultra-processed foods, ad libitum energy intake, and subsequent changes in body weight. Twenty (10 male and 10 female) weight-stable adults (mean ± SE, age = 31.2 ± 1.6 y, BMI = 27 ± 1.5 kg/m^2^) resided in a metabolic ward in the NIH Clinical Center for 28 d (ethical approval was provided by the Institutional Review Board of the National Institute of Diabetes & Digestive & Kidney Diseases [clinicaltrials.gov identifier NCT03407053]). Participants were randomly assigned to receive either an ultra-processed or an unprocessed diet for 2 wk, followed immediately by the alternate diet for another 2 wk. Specific details regarding this study's methodology, including the diet composition of the 2 7-d rotating menus can be found in the Hall et al. (23) article.

In summary, participants were provided with 3 daily meals (breakfast, lunch, and dinner) plus snacks; however, our secondary analysis focuses only on the data from the meals. The 2 diets were matched for a variety of characteristics: total calories, energy density (including beverages), macronutrients, fiber, sugars, and sodium. However, the meals differed in their level of processing based on the NOVA classification scheme (25). Additionally, the participants rated the diets as equally pleasant and familiar, and the 3 daily meals plus snacks were provided in large portions (twice the individual's estimated energy requirements for weight maintenance). Importantly, on average, the “presented meals” (i.e. the meals served to the participant) differed in their nonbeverage ED based on diet type (ultra-processed meals: 1.96 kcal/g, unprocessed meals: 1.06 kcal/g). This provided the rare opportunity to assess ad libitum energy intake across a broad and continuous range of EDs using familiar foods in a highly controlled environment.

### Secondary analysis of the Hall et al. data set on food ultra-processing and energy intake (kcal)

To determine whether the relation between meal ED and meal energy intake is linear (as would be predicted if people did not compensate for energy content by changing meal size [g]), the consumed meal caloric intake (kcal), meal size (g), and ED of each meal was calculated. Meals were collapsed across diet types (i.e. unprocessed or ultra-processed), and we excluded meals that were “plate cleaned” (i.e. >95% of the served portion was consumed) (*n* = 159 meals) and all calorie and noncalorie containing beverages. In a few meals (5% of meals served to each participant), cereal or oatmeal were presented alongside milk. In these cases, we did not exclude milk because neither cereal nor oatmeal were consumed without milk. However, we cannot rule out the possibility that a proportion of the milk was consumed separately, as a beverage.

To control for both individual (participant level) and “meal type” (breakfast, lunch, and dinner) differences in energy intake, we mean centered meal caloric intakes for each participant and for each meal type across the 28 d (20 participants × 3 meal types × 28 d = 1680 total centered meals). So, for example, for participant 1, there were 28 centered meal caloric intakes for “breakfast,” 28 centered meal caloric intakes for “lunch,” and 28 centered meal caloric intakes for “dinner.” Centered meals with *Z*-scores < or > ± 3.29 were treated as outliers and removed, resulting in a final data set with 1519 meals (**Supplementary Figure 1**).

### Overview of the UK National Diet and Nutrition Survey

The 2000–2001 UK National Diet and Nutrition Survey (NDNS) comprises dietary data obtained between July 2000 and June 2001 (24). The aim of the survey was to provide a cross-sectional record of the eating habits and nutritional status of the UK population. A multi-stage random-probability design was used to invite participants; 152 postal sectors were selected during the first stage, and from each sector, 40 addresses were randomly chosen. Individuals who were neither pregnant nor breastfeeding, and those aged between 19 and 64 y were eligible for inclusion. All provided written informed consent and the NDNS received ethical approval from a Multi-center Research Ethics Committee (MREC) and National Health Service Local Research Ethics Committees (LRECs).

### Analysis of data from the UK NDNS

Participants (*N* = 1724; 958 females, 766 males; mean ± SE, age = 42.10 ± 0.29 y; BMI = 26.83 ± 0.13) used a diet diary to record all of the food and drink that they consumed over 7 d. For eating events occurring at home, each food item was individually weighed and recorded, and any uneaten food was subtracted from the initial portion. For out-of-the-home eating events, participants recorded approximate amount or quantities served, and noted any leftovers. We removed all calorie and noncalorie containing beverages using a purpose-written script in R which excluded beverages using food codes from the User Guide provided by the UK Data Service. Remaining beverages which were not identified in the User Guide were manually removed by the lead author on 5 separate instances. We did not exclude milk when it was consumed with cereal or porridge or water when it was used to prepare a powdered soup. Lastly, we calculated the consumed eating event size (g), eating event caloric intake (kcal), and eating event ED for each of the 60,777 recorded eating events.

Our aim was to make the NDNS data set comparable to the Hall et al. data set which only included data from meals. We therefore excluded eating events where <200 kcal had been consumed and where the eating event ED was >4 kcal/g (26). The 200 kcal cut-off corresponds with previous research using the NDNS data set suggesting the average caloric content of snacks to be ∼200 kcal (27). The NDNS provides no information about meal type (i.e. breakfast, lunch, or dinner) and so we use the term “meal” to refer to all eating events that were not excluded. Meal caloric intakes were mean centered within each individual and centered meals with *Z-*scores < or > ± 3.29 were removed from the analyses. The final data set comprised 32,162 meals (**Supplementary Figure 2**).

### Statistical analysis

Initially, we plotted centered meal caloric intakes by consumed meal ED for visual inspection of any evidence for nonlinearity and the remaining analyses were conducted in the R statistical environment (28) with several helper packages (29, 30). To quantify whether a nonlinear fit may better explain the data, a Ramsey Regression Equation Specification Error Test (RESET) was conducted following the procedure outlined by Ramsey (31) and using the R package “lmtest” (32). If the Ramsey RESET returned a significant result, then a segmented regression was run on the centered meal caloric intake data following the procedure described by Muggeo (33) and using the R package “segmented” (34). A segmented regression or “broken stick” regression is an iterative approach which establishes the existence of 1 or multiple breakpoints. First, a simple linear model (without a breakpoint) is computed and evidence for a breakpoint is assessed. If a breakpoint is identified (*P* < 0.05), then a segmented regression is used to establish the location. The process then repeats until no further breakpoints are identified. This approach also constrains the segments to be “continuous” (adjacent regression lines begin and end at the same location) (33). To confirm that a segmented fit is superior to a linear fit, we used the Akaike's and the Bayesian information criterion (“stats” package, [28]).

## Results

### Secondary analysis of the Hall et al. data set: evidence for a nonlinear association between consumed meal ED (kcal/g) and mean centered meal caloric intake (kcal) in a controlled setting

**Figure 1:**
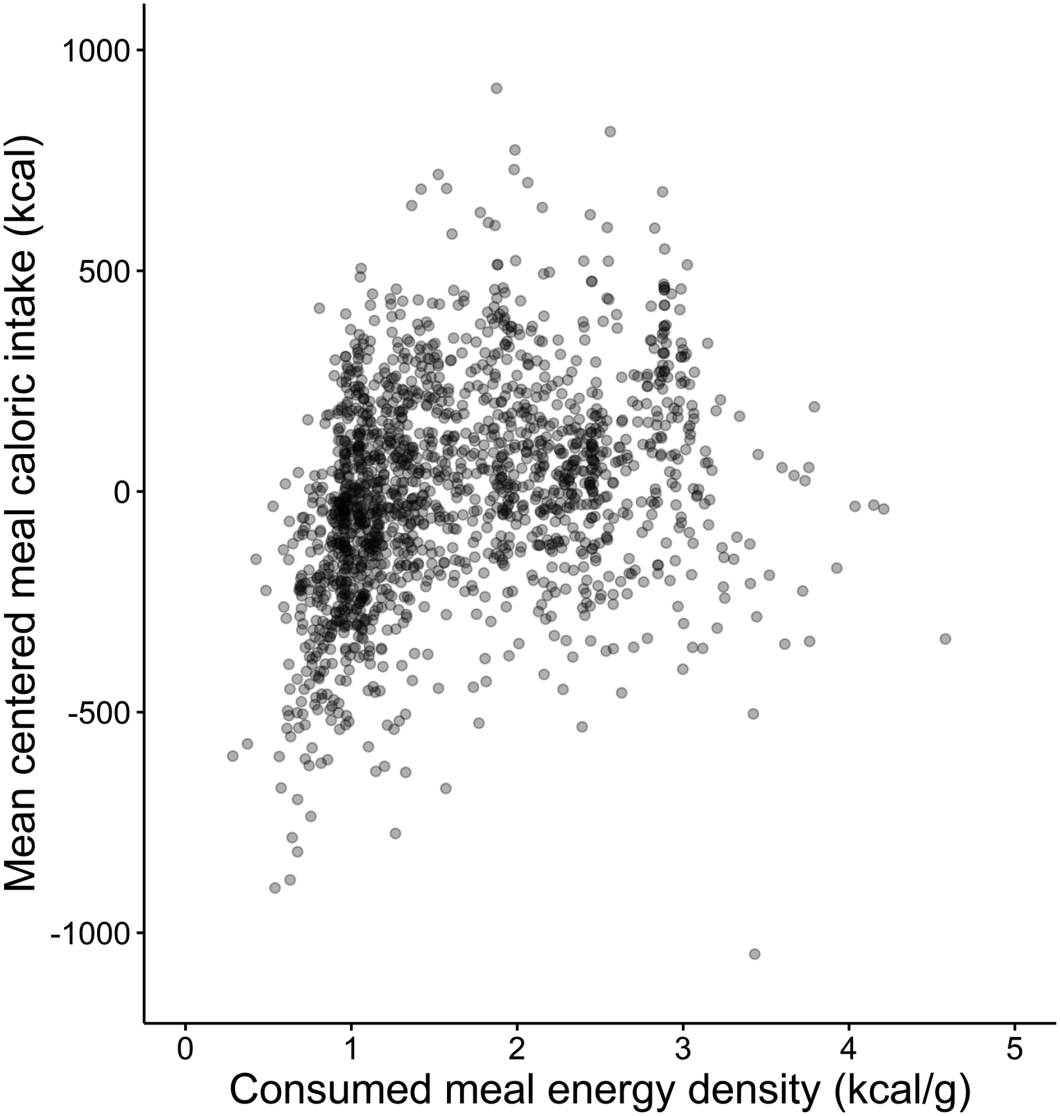
Centered meal caloric intake (kcal) by consumed meal energy density (kcal/g) in the Hall et al. data set *(n* = 1519). Meals were centered within each participant and meal type. Meals which were plate cleaned (i.e. more than 95% of the served portion consumed) and meals with *Z*-scores < or > ± 3.29 were removed. In this scatterplot, each point represents 1 meal.

**Figure 2:**
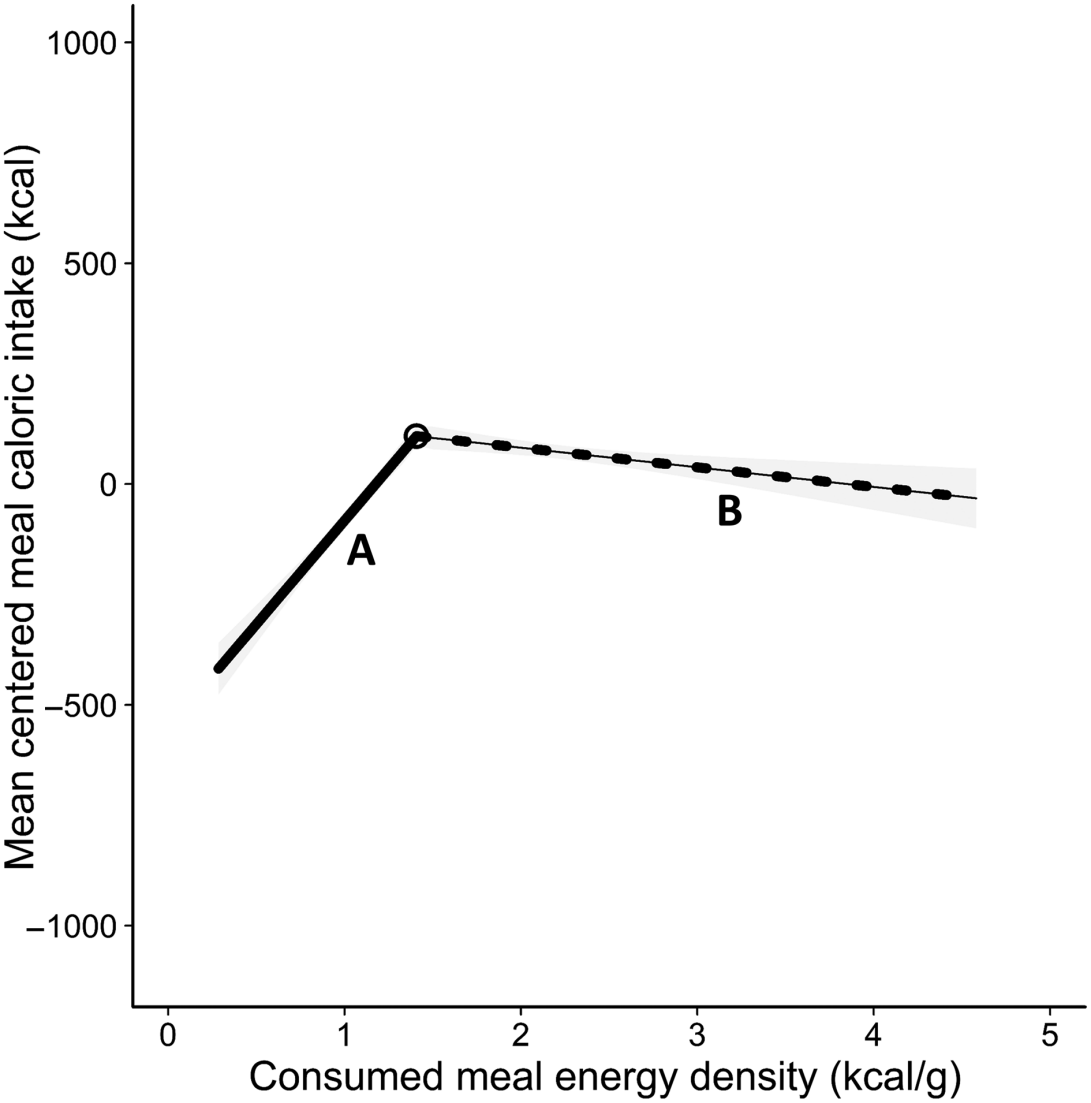
Mean centered meal caloric intakes (kcal), predicted from a segmented regression model relating consumed meal energy density (kcal/g) to consumed centered meal caloric intake (kcal) in the Hall et al. data set *(n* = 1519). The breakpoint located at 1.41 kcal/g (SE = 0.04) is represented by a circle. The dashed and solid lines represent different segments and the shading around the segments indicates 95% CIs. Segment A indicates the slope of the segment below the breakpoint (1.41 kcal/g), and segment B models the slope above the breakpoint (1.41 kcal/g).

Visual inspection of the plot containing centered meal caloric intake by consumed meal ED (**Figure 1**) indicated a potential nonlinear pattern in the data. Centered meal caloric intake appeared to increase with increasing ED until ∼1.5 kcal/g and then decreased slightly. The Ramsey RESET (*F*[2, 1515] = 99.32, *P* < 0.001) indicated that a nonlinear fit would better explain the data, and the segmented regression returned a 1-breakpoint solution (“0 compared with 2,” *P* < 0.001; “1 compared with 2,” *P* = 0.08) at 1.41 kcal/g (SE = 0.04), demonstrating a significant change in the relation between consumed meal ED and centered meal caloric intake at this point (**Table 1**, **Figure 2**). Respectively, we observed a significant positive and negative association below and above 1.41 kcal/g (Table 1). Tests of the Akaike's and Bayesian information criterion supported a segmented fit **(Supplementary Table 1)**. We also noted that the increase in centered meal caloric intake before the 1.41 kcal/g breakpoint and subsequent decrease after the breakpoint was also observed in the raw meal caloric intake data (**Supplementary Figure 3)**. To assess the robustness of this evidence for nonlinearity, we conducted sensitivity analyses, once including *1*) plate cleaned meals and again using *2*) presented meal ED (to account for possible spurious correlations between consumed meal ED and centered meal caloric intake). Here, for both analyses, 2-breakpoint solutions were returned *1*) 1.08 and 2.89 kcal/g and *2*) 1.02 and 1.84 kcal/g (**Supplementary Figures 4 and 5**). Regardless, evidence for nonlinearity was preserved.

**Table 1:** Slope parameter estimates, 95% CIs, *t* values, and *P* values from a segmented regression model predicting centered meal caloric intake (kcal) from consumed meal energy density (kcal/g) in the Hall et al. data set (*n* = 1519)

	Slope parameter	95% CI	*t* value	*P* value
Slope 1 (<1.41 kcal/g)	469.13	396.58, 541.67	13.19	<0.001
Slope 2 (>1.41 kcal/g)	–44.42	–71.94, –16.90	–3.02	0.003

### UK NDNS data set: similar results in participants in free-living conditions

**Figure 3:**
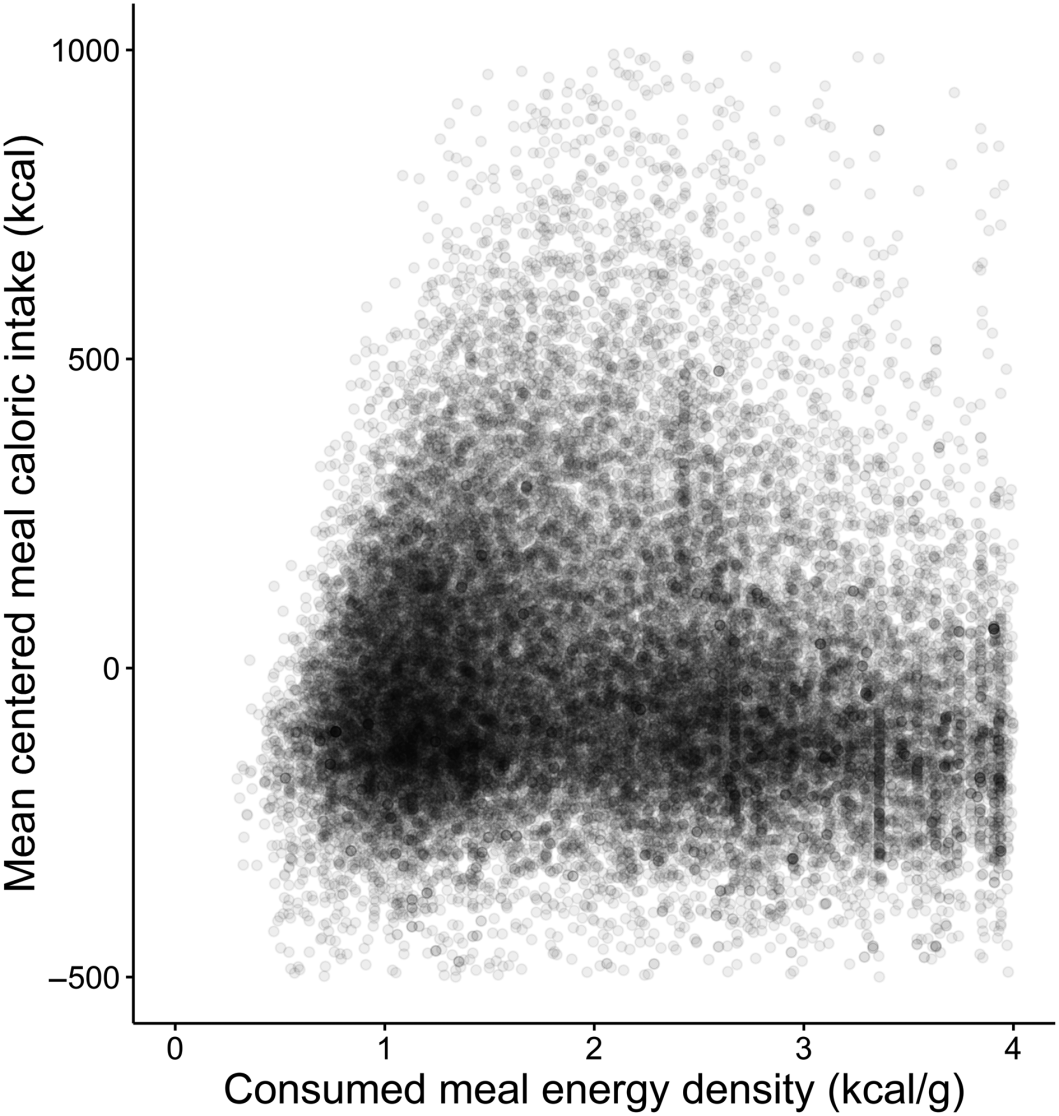
Centered meal caloric intake (kcal) by consumed meal energy density (kcal/g) in the NDNS data set *(n* = 32,162). Meals were centered within each participant and meals with *Z*-scores < or > ± 3.29 were removed. In this scatterplot, each point represents 1 meal. To aid graphical illustration, centered meal caloric intakes above 1000 kcal or below –500 kcal are excluded from this figure (0.51% of total meals). They were, however, included in the reported analyses. NDNS, UK National Diet and Nutrition Survey.

**Figure 4:**
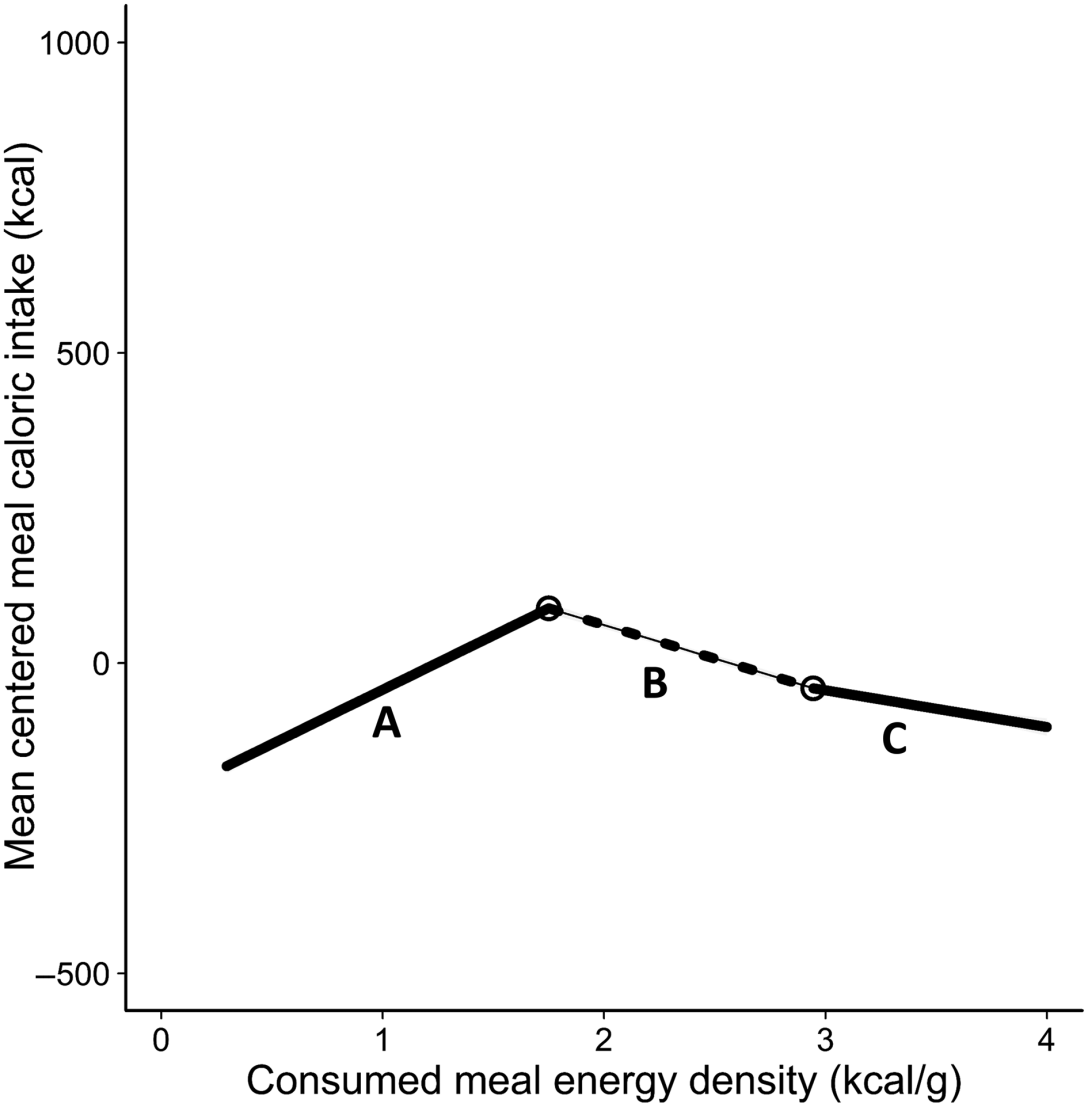
Mean centered meal caloric intakes (kcal), predicted from a segmented regression model relating consumed meal energy density (kcal/g) to consumed centered meal caloric intake (kcal) in the NDNS data set *(n =* 32,162). The breakpoints located at 1.75 kcal/g (SE = 0.02) and 2.94 kcal/g (SE = 0.15) are represented by circles. The dashed and solid lines represent different segments and the shading around the segments indicates 95% CIs. Segment A indicates the slope of the segment below the first breakpoint (1.75 kcal/g), segment B indicates the slope of the segment between the 2 breakpoints (1.75 kcal/g & 2.94 kcal/g), and segment C models the slope above the second breakpoint (2.94 kcal/g). NDNS, UK National Diet and Nutrition Survey.

**Figure 3** shows centered meal caloric intake by consumed meal ED. Unlike in Figure 1, the large number of superimposed datapoints made it difficult to determine nonlinearity and potential breakpoints from simple visual inspection. However, the Ramsey RESET test demonstrated a nonlinear pattern (*F*[2, 32,158] = 852.77, *P* < 0.001), and 2 breakpoints (“0 compared with 2,” *P* < 0.001; “1 compared with 2,” *P* = 0.046) were identified at 1.75 kcal/g (SE = 0.02) and 2.94 kcal/g (SE = 0.15), respectively (**Table 2**, **Figure 4**). Again, we observed a significant positive association below the first breakpoint as well as a negative association between the breakpoints and above the second breakpoint (Table 2), and both the Akaike and the Bayesian information criterion were met (Supplementary Table 1). Regardless of the calorie cut-off used for the inclusion criteria (e.g. meal caloric intake > 600 kcal), the patterns of results were broadly similar; meal caloric intake increased until the first breakpoint and then decreased. A 2-breakpoint solution was returned when the inclusion criterion for meals was set at both 400 and 600 kcal (i.e. meals <400 or 600 kcal were excluded). However, when the criterion was set at 800, 1000, and 1200 kcal a 1 breakpoint solution was returned. Regardless of whether a 1 or 2 breakpoint solution was selected, the first breakpoint occurred between 1.75 kcal/g and 2.30 kcal/g (**Supplementary Table 2**).

**Table 2:** Slope parameter estimates, 95% CIs, *t* values, and *P* values from a segmented regression model predicting centered meal caloric intake from consumed meal energy density in the NDNS data set (*n* = 32,162)^1^

	Slope parameter	95% CI	*t* value	*P* value
Slope 1 (<1.75 kcal/g)	174.86	162.78, 186.94	31.60	<0.001
Slope 2 (1.75 kcal/g – 2.94 kcal/g)	–107.91	–118.99, –96.84	–15.54	<0.001
Slope 3 (>2.94 kcal/g)	–59.19	–81.12, –37.26	–5.56	<0.001

1 NDNS, UK National Diet and Nutrition Survey.

## Discussion

### Evidence for sensitivity to ED

Evidence for a nonlinear association between centered meal caloric intake and consumed meal ED was found in both the Hall et al. and the UK NDNS data sets. Centered meal caloric intake increased with ED until the first breakpoint (segment A) and decreased thereafter (segment B, and, in the NDNS data set, segment C [Figure 2 & Figure 4]). It is axiomatic that the observed trends in caloric intake resulted from participants consuming different sized meals (g) across the ED range. In segment A of both data sets, participants consumed similar sized meals (**Supplementary Figure 6 and 7**), resulting in the positive association between consumed ED and centered meal caloric intake. That is, there was no indication that participants compensated for the increasing ED by reducing their meal size. However, the negative slopes in segment B of both data sets and segment C of the NDNS data set reflect a degree of overcompensation; specifically, participants consumed smaller meals than necessary to adjust for the increasing meal ED. The overcompensation may, in part, be driven by cognitive restraint, specifically the conscious restriction of meal size due to concerns about effects of energy-rich meals on body weight (27). This overcompensatory reduction in the meal size of high energy-dense foods is important in curbing overall energy intake, given that meals in the NDNS data set with an ED above the first breakpoint (1.75 kcal/g) contribute ∼60% of total energy intake (**Supplementary Figure 8**).

In summary, had participants been insensitive to meal energy content, then they would have eaten the same amount of food resulting in a linear increase in centered meal caloric intake with ED (we found the converse). It should be acknowledged that the patterns are not identical across data sets, which may reflect differences in study populations (US compared with UK) or study conditions (controlled compared with free-living). Nevertheless, we see evidence for overcompensation in higher energy-dense meals in both data sets. Future research should repeat these analyses in data sets from other countries.

### A 2-component model of meal size (g): “volume” and “calorie-content” satiation signals

**Figure 5:**
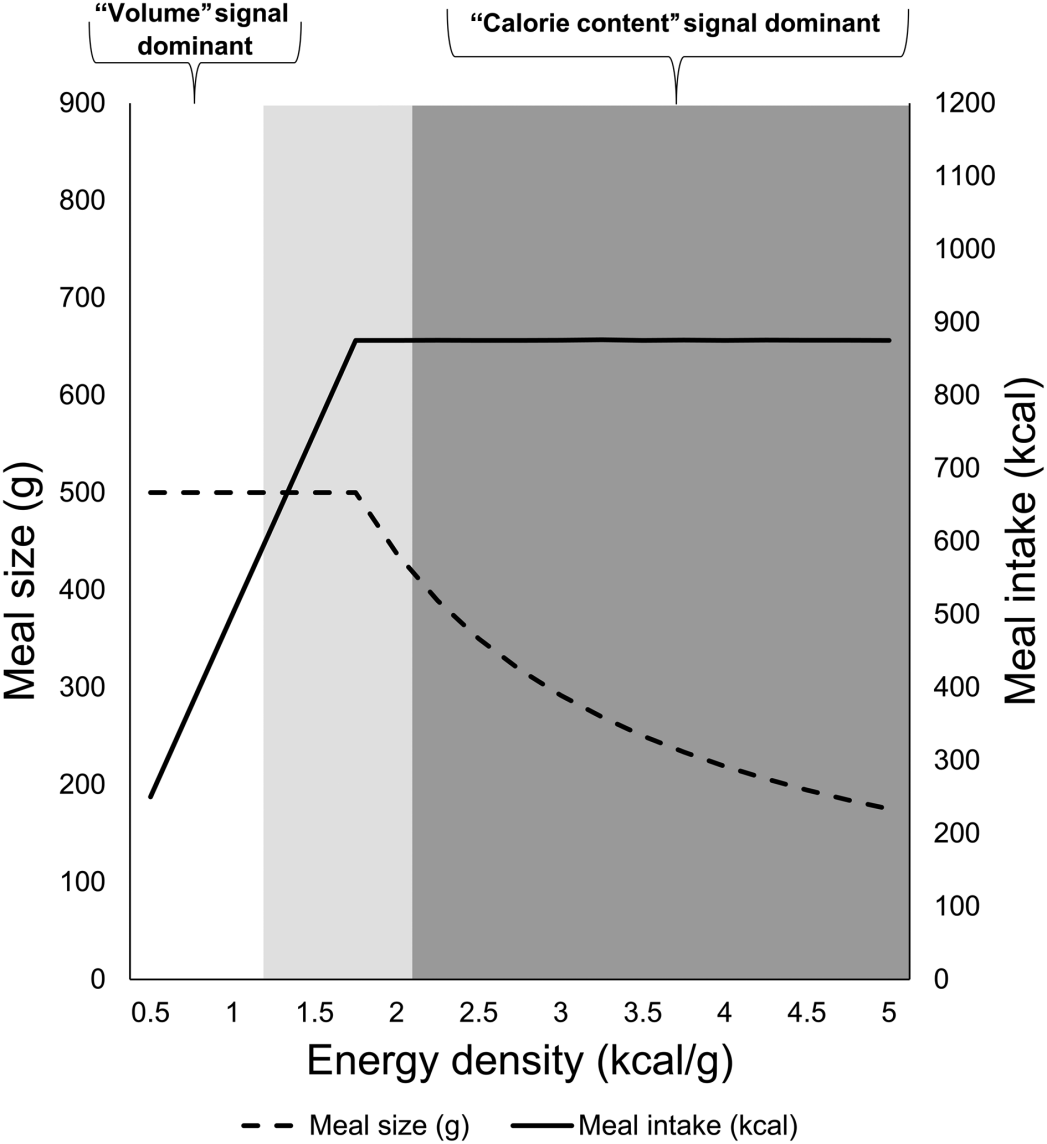
Two-component model of meal size (g): volume and calorie-content satiation signals. This is modeled using an 875 kcal meal as an example and demonstrates perfect compensation. The white section indicates the dominance of the “volume” signal, the dark gray section the dominance of the “calorie-content” signal, and the lighter gray section indicates where a breakpoint might occur which is the location where the relative dominance of the signals changes.

These findings can be captured by a 2-component (“volume” and “calorie content”) model of meal size (similar to Smith [35] and Deutsch [36]) in which volume is the dominant signal with energy-dilute foods and calorie content is the dominant signal with energy-rich foods (**Figure 5**). The volume signal is a largely unconditioned response that affects food intake via gastric distension, whereas the calorie-content signal (biologically derived from the sensing of fat, carbohydrate, and protein) reduces meal size based on learned (anticipatory) and unlearned (immediate) effects of calories. Both the volume and calorie-content signals can impact meal size via food portion-size selection (expected satiety) (37, 38) or within a meal directly (39, 40). Although feedback from the volume signal is constant across a range of EDs, it is more salient with lower energy-dense meals. Low energy-dense foods are relatively dilute in calories and are high in intracellular water content and fiber (41). This means that there is little feedback from the calorie-content signal, so the primary determinant of meal size is negative feedback from the volume signal via gastric distension.

With respect to the above model, which posits 2 signals influencing meal caloric intake, we can apply the following reasoning. In both data sets, the positive association between ED and centered meal caloric intake observed in the lower energy-dense meals (i.e. segment A) was driven by participants consuming a similar sized meal across the range of EDs. Thus, it would appear that feedback from the volume signal reached a tolerable upper limit, capping lower energy-dense meals at a similar size.

With respect to the calorie-content signal, frequently consuming a food provides the opportunity to learn from delayed postingestive experiences (e.g. malaise if overconsumed) (42, 43). High energy-dense foods will provide relatively greater postingestive caloric feedback via the calorie-content signal. For the signal to operate effectively, it is critical that the usual relation between taste and calorie content is preserved. This allows participants to use previous postingestive experiences to guide the amount of food consumed either via premeal planning (expected satiety) (44) or during a meal via “conditioned satiation” (39, 40). Importantly, the reduction in meal size in response to increasing ED (segments B and C) is not only present at an individual level, but might also be reflected on a larger scale, such as in full-service and fast-food restaurant meals (45).

Finally, the success of the 2-component model is that it explains complexity in the relation between ED, meal size, and energy intake. However, it is not exhaustive, and does not consider a role for individual macronutrients or effects of moderators such as eating rate (46) and appetition (47).

### Reconciling ad libitum and preload test-meal studies with the 2-component model

Short-term ad libitum studies (e.g. <10 total exposure days) report ED has little to no influence on meal size. Based on the model, this insensitivity is seen for 2 reasons. First, ED is often manipulated covertly, which undermines the learned calorie-content signal. Second, the meals or diets are often energy dilute (e.g. <2 kcal/g), which means the volume signal dominates. In combination, this explains the tendency to consume a consistent weight of food in many ad libitum studies. Indeed, it has been previously observed that this tendency might only occur below a certain low ED (48) and the *Volumetrics Eating Plan* (49) illustrates how this strategy can generate sustained weight loss (50).

By contrast, preload test-meal studies demonstrate some sensitivity to food ED and reflect an unconditioned calorie-content signal. Here, an interval exists between the preload and the test meal. Therefore, the calorie content of the preload, even when covertly manipulated, can be detected (by the gut, e.g. Wilbrink et al. [51]) to effect subsequent test-meal intake. Moreover, for longer-term ad libitum studies, the effects of ED on meal size could be explained by the capacity of the calorie-content signal to influence satiation indirectly, via associative learning. Specifically, the orosensory features of the food become associated with the postingestive consequences of its calorie (macronutrient) content which, over time, come to modify meal size, a phenomenon similar to “expected satiety” (38, 52).

### Differences in sensitivity to ED in food choice and food intake

It may seem paradoxical that there are contrasting patterns of sensitivity to ED in studies of food choice and food intake. In choice studies, a clear linear association (positive) is observed between ED and preference, but only in lower energy-dense foods (∼ <1.75 kcal/g) (22, 53). In foods with progressively higher ED (∼ >1.75 kcal/g), this relation weakens until choice and ED become unrelated (22). Whereas for food intake, the present results demonstrate the converse – greater sensitivity to ED in higher ED meals.

These different findings may reflect an adaptation that maximizes caloric intake in an environment in which ED varies substantially, while at the same time avoiding the acute aversive effects of short-term overconsumption (42, 54). Differences in the ED of energy-dilute foods matter because stomach capacity is limited. When only energy-dilute foods are available, choosing the least energy-dilute (most energy-dense) food will ensure that energy intake is maximized. By contrast, with energy-rich options, absolute stomach capacity is relatively unimportant, and the priority shifts to avoiding acute, negative soporific effects caused by an overconsumption of calories (42). Accordingly, we observe a compensatory reduction in meal size with ED, which as noted above, is driven by a largely learned anticipation of the effects of the food's calories on satiety (i.e. the calorie-content signal).

### Conclusion

As ad libitum and preload test-meal studies report contrasting findings regarding the effects of ED on energy intake, we explored this association in 2 data sets. Uniquely, we measured the influence of meal ED on meal energy intake: *1*) across a broad and continuous range of EDs, *2*) using noncovertly manipulated, “real-world” foods, and *3*) allowed for the possibility that the association is nonlinear. We observed a consistent nonlinear pattern which we explain using a 2-component model comprising volume and calorie-content signals. This model also reconciles differing results in ad libitum and preload test-meal studies investigating covert manipulation of meal ED. Indeed, our findings challenge the idea of “passive overconsumption” of energy-rich foods and show that, by contrast, surprisingly, people show overcompensation.

## Supplementary Material

nqac112_Supplemental_File

## Data Availability

All analytic code and summary data from the Hall et al. study will be made available upon request. UK NDNS data were obtained from the UK Data Service and can be accessed via application to and approval by the UK Data Service.

## Notes

KDH and ABC are supported by the Intramural Research Program of the National Institutes of Health, National Institute of Diabetes & Digestive & Kidney Diseases.

Supplementary Figures 1–8 and Supplementary Tables 1 and 2 are available from the “Supplementary data” link in the online posting of the article and from the same link in the online table of contents at https://academic.oup.com/ajcn/.

Abbreviations used: ED, energy density (kcal/g), NDNS, UK National Diet and Nutrition Survey; RESET, Regression Equation Specification Error Test.
