# Supplementary material for: Time to revisit the passive overconsumption hypothesis? Humans show sensitivity to calories in energy-rich meals
Source: Am J Clin Nutr. 2022 Apr 30;116(2):581–8. doi: 10.1093/ajcn/nqac112 (PMC9348985; doi:10.1093/ajcn/nqac112)
Supplement: nqac112_Supplemental_File [file nqac112_supplemental_file.pdf]

Table of Contents

Supplementary Table 1 2
Supplementary Table 2 3
Supplementary Figure 1 4
Supplementary Figure 2 5
Supplementary Figure 3 6
Supplementary Figure 4 7
Supplementary Figure 5 8
Supplementary Figure 6 9
Supplementary Figure 7 10
Supplementary Figure 8 11

Supplementary Table 1

Akaike's information criterion value, Bayesian information criterion value and degrees of freedom for both a linear and a segmented regression model in the Hall (*n* = 1,519) *and NDNS (n = 32,162) datasets.*

	Degrees of freedom	Akaike's information criterion value	Bayesian information criterion value
Hall dataset			
Linear	3	20673	20689
Segmented	5	20485	20511
NDNS dataset			
Linear	3	440532	440557
Segmented	7	438829	438888

Supplementary Table 2

Breakpoints and slopes from a segmented regression model in the NDNS dataset (n = 32,162) using various calorie filters (i.e., eating events excluded if less than specified calorie value): if two breakpoints were not identified as being significant via the segmented regression, then only one breakpoint is reported.

Calorie filter (kcal)	Breakpoint 1	Breakpoint 2	Slope 1 (Segment A)	Slope 2 (Segment B)	Slope 3 (Segment C)
200	1.75	2.94	174.86	-107.91	-59.19
400	1.84	2.89	183.95	-112.17	-34.85
600	2.04	2.66	155.28	-151.12	0.33
800	1.77		184.85	-18.38	
1000	1.95		173.64	-25.40	
1200	2.30		116.04	-79.79	

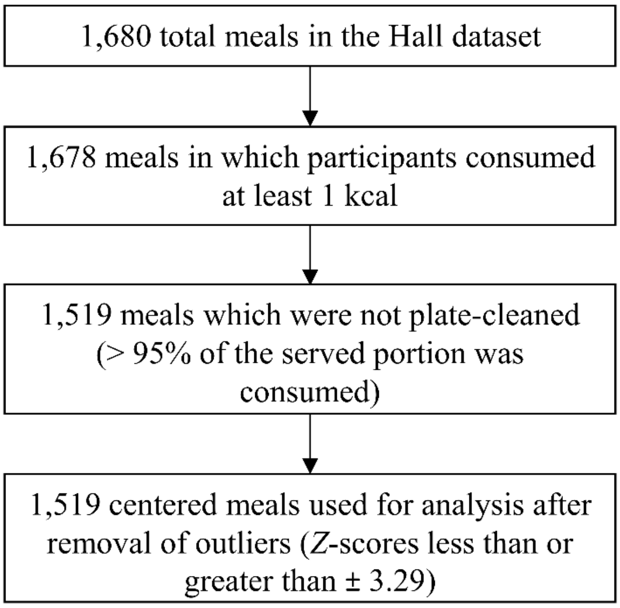

Supplementary Figure 1 Flow of meals from the Hall dataset through the exclusion stages.

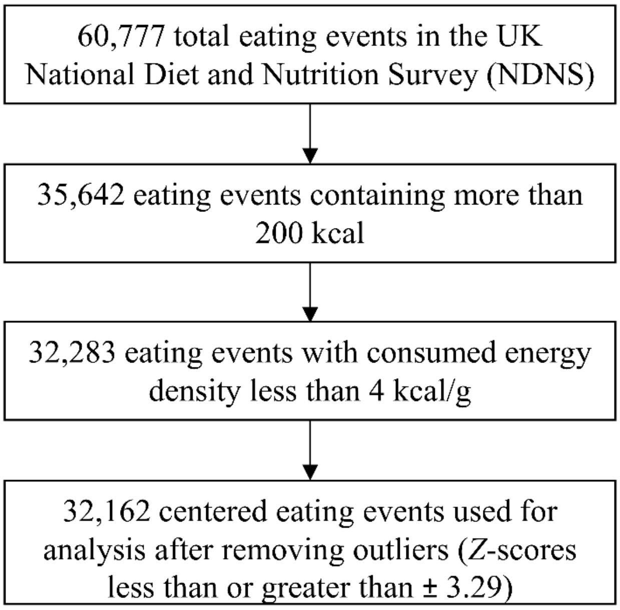

Supplementary Figure 2 Flow of eating events from the NDNS dataset through the various exclusion stages.

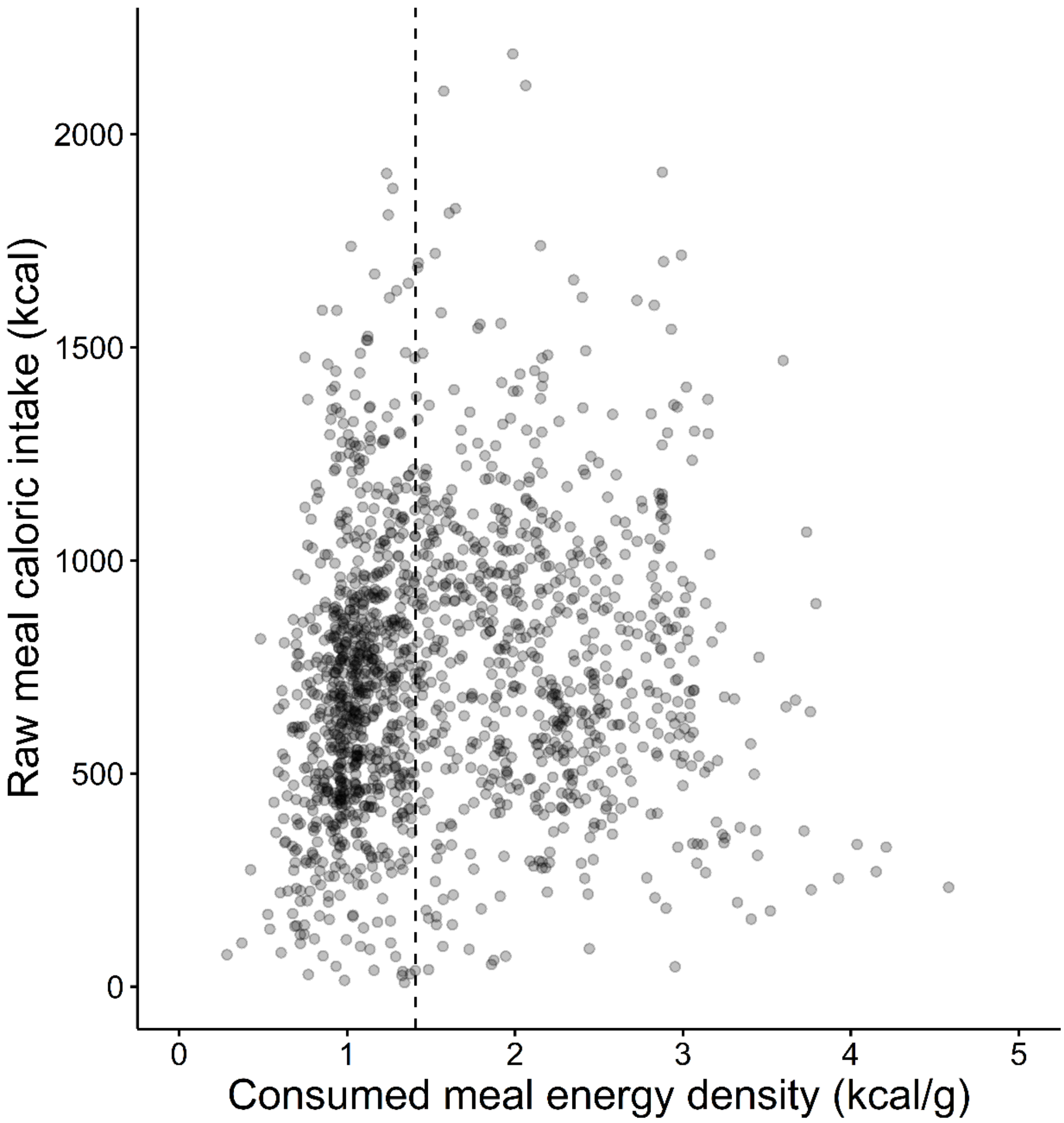

Supplementary Figure 3 Raw meal caloric intake (kcal) by consumed meal energy density (kcal/g) in the Hall dataset (*n* = 1,519). Z-scores of raw meal caloric intake values were calculated, and outliers with Z-scores less than or greater than ± 3.29 were removed. The black dashed line represents the 1.41 kcal/g breakpoint identified via segmented regression. In this scatterplot, each point represents one meal.

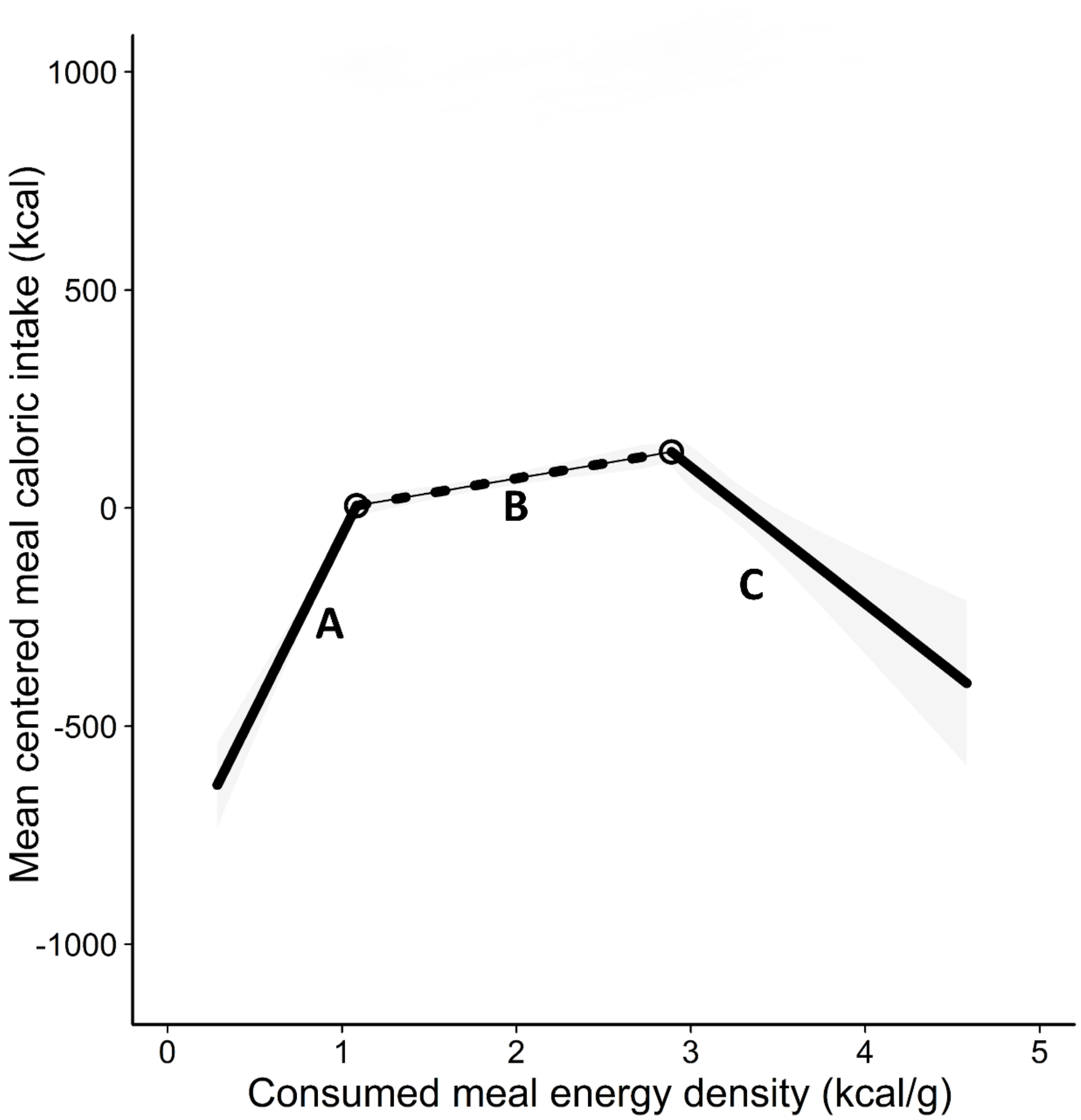

Supplementary Figure 4 Mean centered meal caloric intakes (kcal), predicted from a segmented regression model relating consumed meal energy density (kcal/g) to consumed centered meal caloric intake (kcal) in the Hall dataset *(n* = 1,678) including plate cleaned meals (i.e., > 95% of the served portion was consumed). The dashed and solid lines represent different segments and the shading around the segments indicates 95% confidence intervals. The circles indicate the location of significant breakpoints at 1.08 kcal/g (*SE* = 0.03) and 2.89 kcal/g (*SE* =0.08). Segment A indicates the slope of the segment between 0 and 1.08 kcal/g, segment B indicates the slope of the segment between 1.08 and 2.89 kcal/g, and segment C models the slope above 2.89 kcal/g.

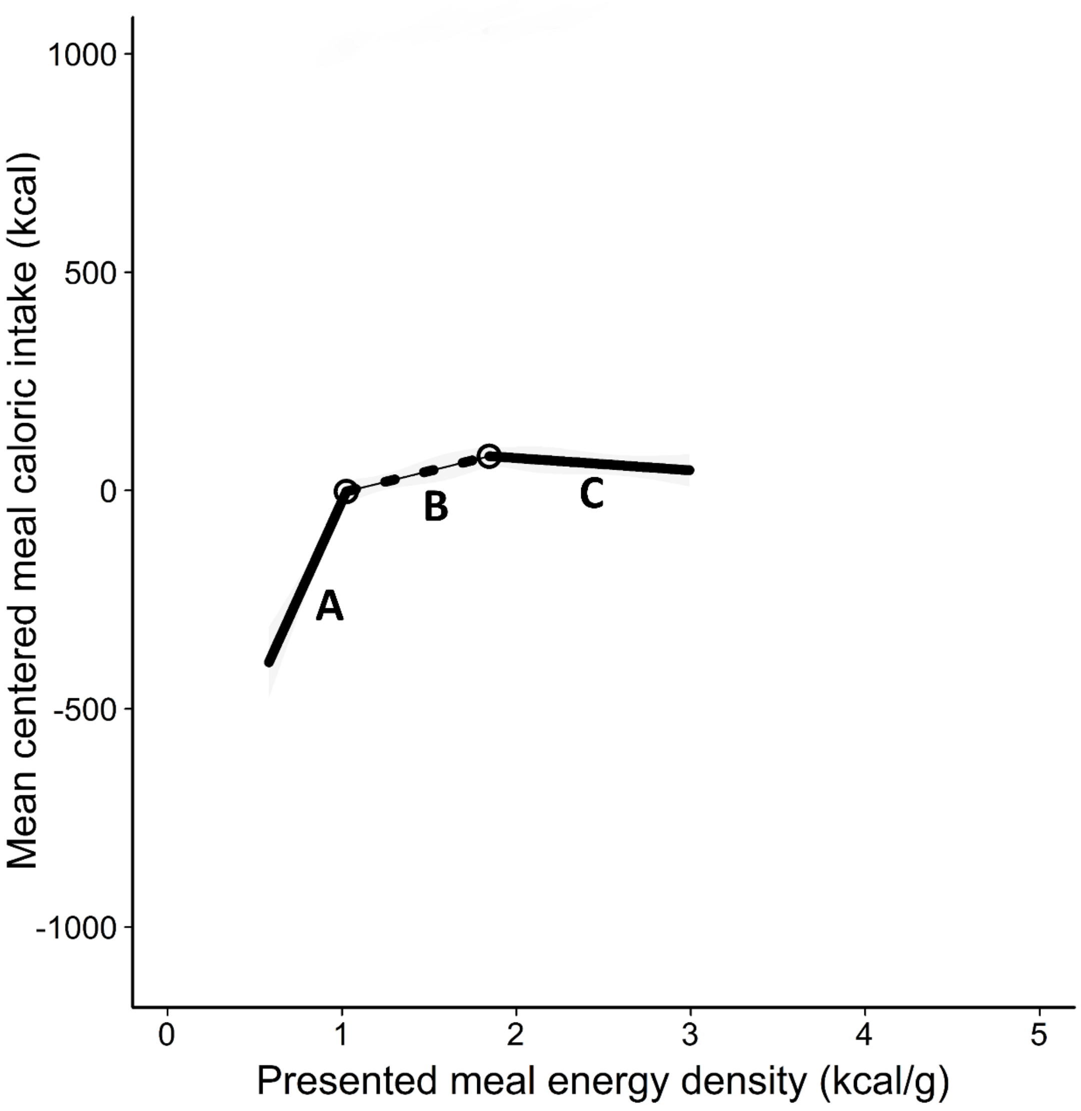

Supplementary Figure 5 Mean centered meal caloric intakes (kcal), predicted from a segmented regression model relating presented meal energy density (kcal/g) to consumed centered meal caloric intake (kcal) in the Hall dataset *(n* = 1,519). The dashed and solid lines represent different segments and the shading around the segments indicates 95% confidence intervals. The circles indicate the location of significant breakpoints at 1.02 kcal/g (*SE* = 0.03) and 1.84 kcal/g (*SE* = 0.20). Segment A indicates the slope of the segment between 0 and 1.02 kcal/g, segment B indicates the slope of the segment between 1.02 and 1.84 kcal/g, and segment C models the slope above 1.84 kcal/g.

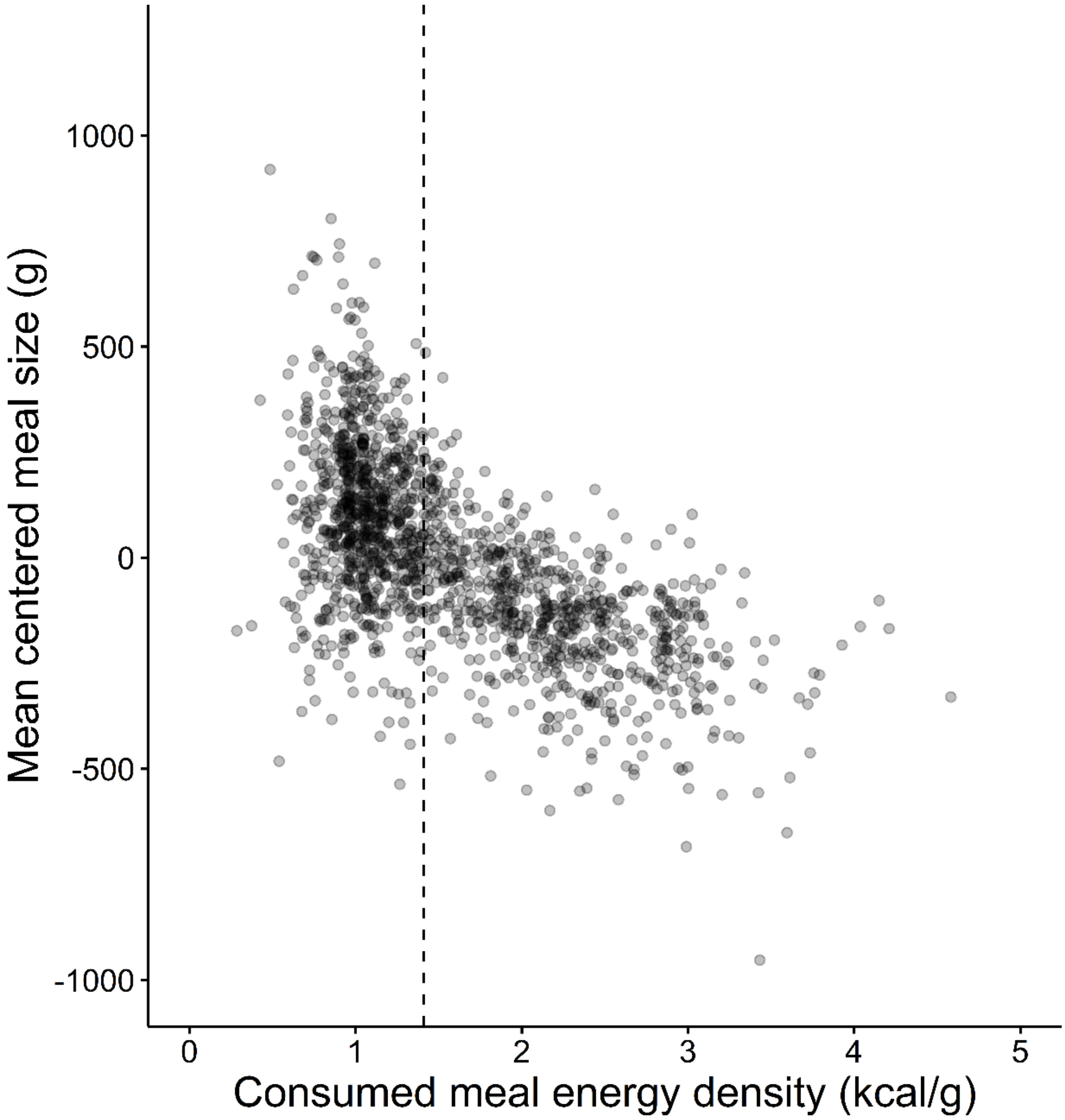

Supplementary Figure 6 Centered consumed meal size (g) by consumed meal energy density (kcal/g) in the Hall dataset (*n* = 1,519). Meals were centered within each participant and meal type and, based on the Z-scores of centered meal caloric intake values, outliers with Z-scores less than or greater than ± 3.29 were removed. In this scatterplot, each point represents one meal. The black dashed line represents the 1.41 kcal/g breakpoint identified via segmented regression.

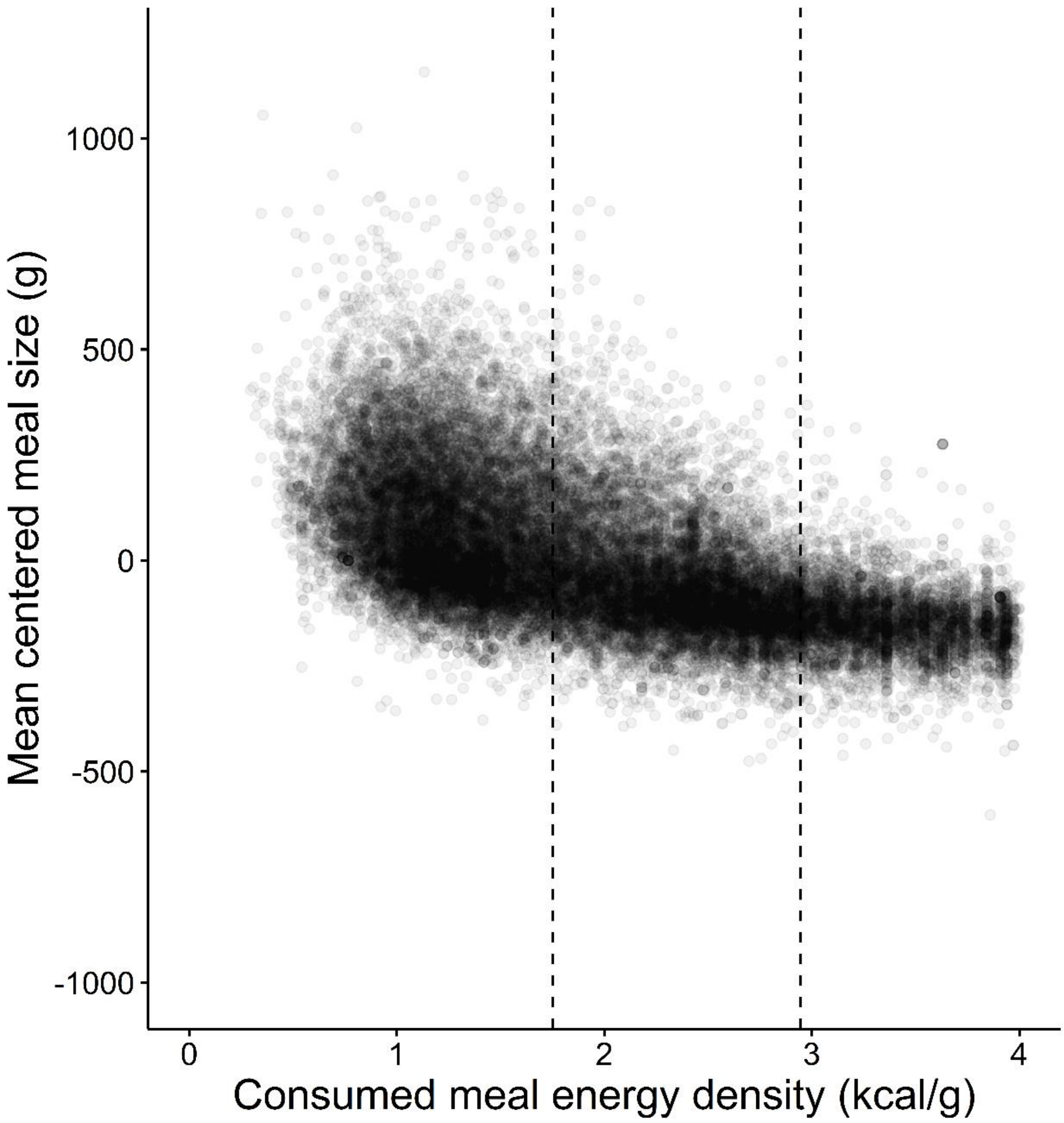

Supplementary Figure 7 Centered consumed meal size (g) by consumed meal energy density (kcal/g) in the NDNS dataset (*n* = 32,162). Meals were centered within each participant and, based on the Z-scores of the centered meal caloric intake (kcal), outliers with Z-scores less than or greater than ± 3.29 were removed. In this scatterplot, each point represents one meal. The black dashed lines represent the 1.75 and 2.94 kcal/g breakpoints identified via segmented regression.

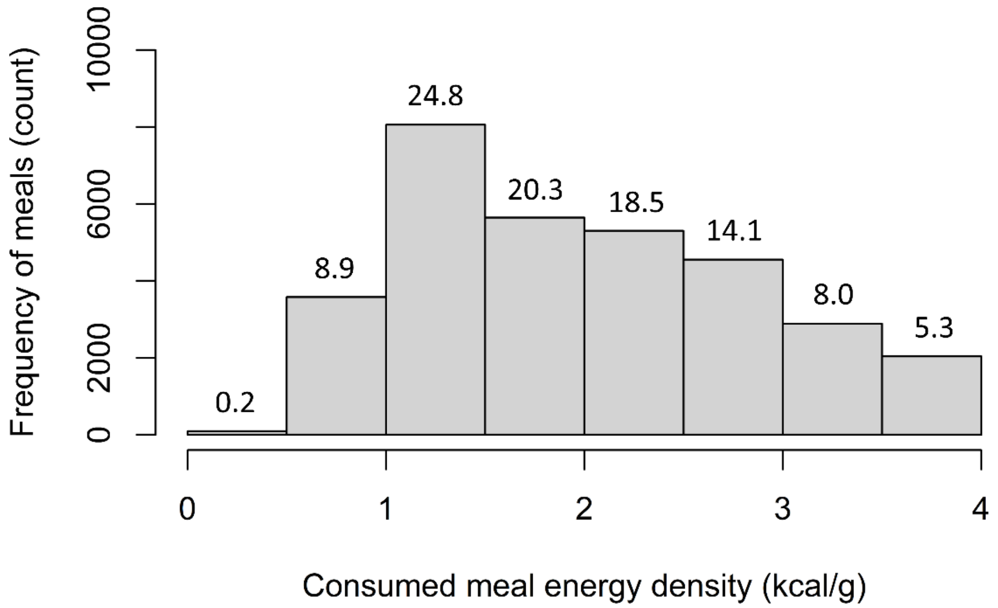

Supplementary Figure 8 Number of meals occurring within each 0.5 energy density (kcal/g) bin in the NDNS dataset (*n* = 32,162). The number at the top of each bar indicates the percentage of total energy intake from meals occurring within each bin.
